# Circulating cell-free DNA as a biomarker for diagnosis of *Schistosomiasis japonica*

**DOI:** 10.1186/s13071-024-06203-x

**Published:** 2024-03-06

**Authors:** Yu Zhang, Rangjiao Liu, Junhui Li, Hongchang Ma, Wenjuan Bao, Jie Jiang, Chen Guo, Deyong Tan, Xing Cheng, Lizhong Dai, Yingzi Ming

**Affiliations:** 1 Transplantation Center, The Third Xiangya Hospital, Central South University, Changsha, Hunan China; 2 Engineering and Technology Research Center for Transplantation Medicine, of National Health Commission, Changsha, Hunan China; 3 Hunan Province Clinical Research Center for Infectious Diseases, Changsha, Hunan China; 4 Sanway Clinical Laboratories, Changsha, Hunan China; 5 Sansure Biotech Incoporation, Changsha, Hunan China

**Keywords:** cfDNA, *Schistosomiasis japonica*, Diagnosis

## Abstract

**Background:**

Schistosomiasis, a neglected tropical disease, remains an important public health problem. Although there are various methods for diagnosing schistosomiasis, many limitations still exist. Early diagnosis and treatment of schistosomiasis can significantly improve survival and prognosis of patients.

**Methodology:**

Circulating cell-free (cf)DNA has been widely used in the diagnosis of various diseases. In our study, we evaluated the diagnostic value of circulating cfDNA for schistosomiasis caused by *Schistosoma japonicum*. We focused on the tandem sequences and mitochondrial genes of *S. japonicum* to identify highly sensitive and specific targets for diagnosis of *Schistosomiasis japonica*.

**Results:**

Through data screening and analysis, we ultimately identified four specific tandem sequences (*TD-1*, *TD-2*, *TD-3*. and *TD-4*) and six mitochondrial genes (*COX1(1)*, *COX1(2)*, *CYTB*, *ATP6*, *COX3*, and *ND5*). We designed specific primers to detect the amount of circulating cfDNA in *S. japonicum*-infected mouse and chronic schistosomiasis patients. Our results showed that the number of tandem sequences was significantly higher than that of the mitochondrial genes. A *S. japonicum* infection model in mice suggested that infection of *S. japonicum* can be diagnosed by detecting circulating cfDNA as early as the first week. We measured the expression levels of circulating cfDNA (*TD-1*, *TD-2*, and *TD-3*) at different time points and found that *TD-3* expression was significantly higher than that of *TD-1* or *TD-2*. We also infected mice with different quantities of cercariae (20 s and 80 s). The level of cfDNA (*TD-3*) in the 80 s infection group was significantly higher than in the 20 s infection group. Additionally, cfDNA (*TD-3*) levels increased after egg deposition. Meanwhile, we tested 42 patients with chronic *Schistosomiasis japonica* and circulating cfDNA (*TD-3*) was detected in nine patients.

**Conclusions:**

We have screened highly sensitive targets for the diagnosis of *Schistosomiasis japonica*, and the detection of circulating cfDNA is a rapid and effective method for the diagnosis of *Schistosomiasis japonica*. The levels of cfDNA is correlated with cercariae infection severity. Early detection and diagnosis of schistosomiasis is crucial for patient treatment and improving prognosis.

**Graphical Abstract:**

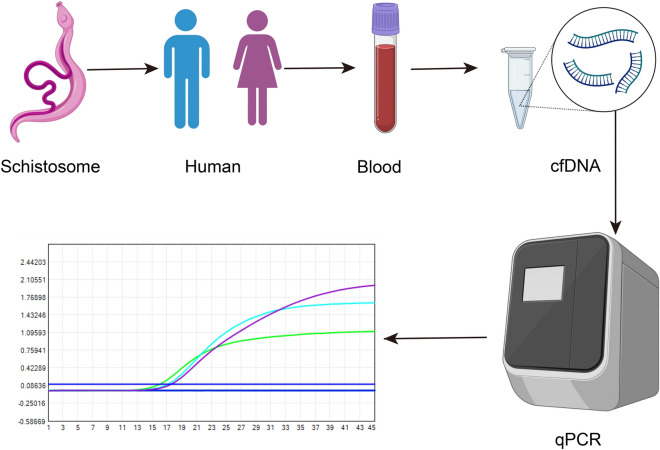

**Supplementary Information:**

The online version contains supplementary material available at 10.1186/s13071-024-06203-x.

## Background

Schistosomiasis is a parasitic disease, also known as bilharziasis, afflicting more than 230 million people worldwide [1]. The main clinical species of *Schistosoma* are *Schistosoma mansoni*, *Schistosoma haematobium*, and *Schistosoma japonicum *[2]. *S. mansoni* and *S. japonicum* cause intestinal and hepatic schistosomiasis, while *S. haematobium* causes urogenital schistosomiasis [3, 4]. After exposure to the cercariae of schistosome, there is acute schistosomiasis syndrome including fever, diarrhea, abdominal pain, fatigue, and malaise [5]. Praziquantel (PZQ) is the preferred drug, which is efficient against adult worms but is inactive against other stages of schistosomes, such as schistosomula and eggs [6]. The chronic schistosomiasis is mainly due to the schistosome eggs deposited in different organs and tissues [7]. Egg-induced granuloma formation and subsequent fibrosis in liver leading to portal hypertension and hepatosplenomegaly is the cause of schistosomiasis-associated mortality [8].

There are various diagnostic techniques available for the diagnosis of schistosomiasis. The most traditional and gold standard diagnostic method is microscopic demonstration of eggs in stool or urine specimens. The Katz–Kato thick smear technique is frequently employed to detect eggs due to its affordability and rapidity [9]. However, because of the random distribution of parasite eggs in stool specimens, it often requires a certain method for egg enrichment. Moreover, the samples examined under a microscope are limited, which often results in false negative results. For patients with acute schistosomiasis, the absence of detectable eggs does not necessarily indicate absence of schistosome infection. There are many methods to detect anti-schistosoma antibodies such as enzyme-linked immunosorbent assay (ELISA), immunofluorescence assay, and indirect hemagglutination assay [10, 11]. Detection of antibodies can confirm infection of schistosome, but it cannot discriminate between active and past infection, which limits the clinical significance of detecting antibodies [12]. Polymerase chain reaction (PCR) of fecal or tissue biopsy samples has higher sensitivity and accuracy for diagnosis of schistosomiasis. However, PCR involves sample extraction of DNA from eggs before amplification, so its limitations are similar to microscopy detection, which depend on whether the sample contains schistosome eggs.

The detection of circulating cell-free DNA (cfDNA) in human plasma, which can be released from necrotic and apoptotic cells, has become increasingly important for diagnosis of different diseases [13]. Recently, circulating cfDNA has been widely used in fields such as oncology, prenatal diagnosis, and organ transplantation [14–18]. Compared with eggs in stool or urine, circulating cfDNA is equally distributed in the blood, solving the problem of random sampling of traditional detection methods. In our study, we screened potential target genes from the gene sequence of *S. japonicum* to investigate the diagnostic role of cfDNA in *S. japonica*.

## Methods

### Human subjects

Peripheral blood was obtained from patients with *S. japonica* and healthy volunteers. We included patients in the study with chronic schistosomiasis who have a history of exposure to contaminated water, positive detection of eggs in their stool, and have received treatment with praziquantel. We had several exclusion criteria including seropositivity for hepatitis B or C virus, alcohol-induced cirrhosis, autoimmune liver or other autoimmune disease, and tumor patients. Our study was approved by the Ethics Committee of the 3rd Xiangya Hospital of Central South University and received written informed consent.

### Mice model of *Schistosomiasis japonica*

Male C57BL/6 mice (6 weeks old) were purchased from Hunan Slack Jingda Experimental Animal Company. Mice with free accessed to food and water were housed under specific pathogen-free conditions at the Experimental Animal Center of Central South University. *S. japonicum*-infected *Oncomelania hupensis* snails were provided by Hunan Provincial Institute of Parasitic Diseases in China. After inducing the release of cercariae, mice were subsequently infected percutaneously with 25 ± 2 freshly shed cercaria. To establish mice with different degrees of infection, mice were infected with 20 cercariae and 80 cercariae, respectively. The mice were sacrificed at different time points. The whole blood of mice was collected at 1 week, 3 week, 5 week, and 8 week postinfection.

### DNA extraction

We collected whole blood anticoagulated with ethylenediaminetetraacetic acid (EDTA), which was centrifuged 20 min at 300 *g*. After obtaining the plasma, it was centrifuged again at 6000 *g* for 5 min to thoroughly remove residual cells and debris. We extracted cfDNA using 500 µL of human plasma or 200 µL of mouse plasma with VAHTS serum/plasma circulating DNA kit (Vazyme, Nanjing, China). The extraction of DNA was performed according to the manufacturer’s instructions. We obtained liver samples from normal mice and from mice infected with cercariae for 8 weeks. The liver DNA extraction was performed using Multi-type sample DNA extraction-purification kit (S1005, Sansure Biotech Incoporation, Changsha, China). The extraction of DNA was also performed according to the manufacturer’s instructions.

### PCR amplification

Real-time quantitative PCR (qPCR) was performed using Taqman probes. The PCR was performed in a total reaction volume of 50 μL containing forward primer of target genes 0.25 μL, reverse primer of target genes 0.25 μL, probe of target genes 0.125 μL, forward primer of GAPDH 0.2 μL, reverse primer of GAPDH 0.2 μL, probe of GAPDH 0.1 μL, Taq DNA Polymerase 1.7 μL, dNTP 0.8 μL, MgCl_2_ 0.15 μL, PCR buffer 35.925 μL, UNG enzyme 0.3 μL, and cDNA 10 μL. The condition for PCR were as follows: 2 min at 50 °C (UNG reaction), 95 °C for 2 min (Taq DNA Polymerase activation), 95 °C for 15 s (denaturation), and 60 °C for 30 s (annealing and extension), followed by 45 cycles. qPCR was performed on the SLAN-96P Real-Time PCR System.

### Quantification standard

The target plasmid was synthesized at Sangon Biotech Co. Ltd and quantified using digital PCR on the QIAcuity Four Platform System from Qiagen. A standard curve was prepared based on plasmid with different concentrations, and the sample concentration was calculated according to the standard curve result (Additional file 1: Fig. S1).

Statistical analyses

The significance level was tested by unpaired *t*-test and Chi-square test. The data are expressed as the means ± s.d. *P*-values < 0.05 were considered statistically significant. The calculations were performed using GraphPad Prism software package 8.0 (GraphPad Prism, San Diego, CA, USA).

## Results

### Screening of diagnostic targets for *Schistosomiasis japonica*

**Fig. 1:**
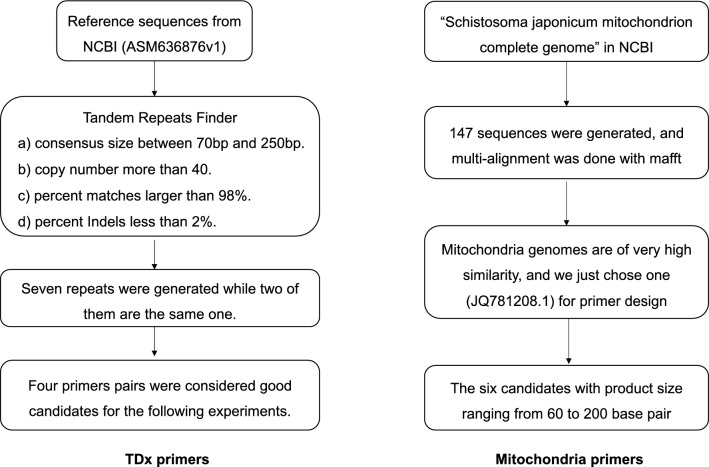
The flowchart for screening candidate targets for diagnosis of *Schistosomiasis japonicum*. Tandem sequences and mitochondrial genes of *Schistosoma japonicum* were focused to identify highly sensitive and specific targets for *Schistosomiasis japonica*. Through data screening and analysis, we ultimately obtained four pairs of tandem primers and six pairs of mitochondrial gene primers

To improve the accuracy of schistosomiasis diagnosis, we strived to identify highly sensitive and specific targets for schistosomiasis japonica. We focused on the tandem sequences and mitochondrial genes of *S. japonicum* (Fig. 1). Tandem sequences refer to short sequences that repeat consecutively in a gene or DNA fragment. We downloaded genomes sequences of *S. japonicum* from the National Center for Biotechnology Information (NCBI, ASM636876v1), and scanned the repeat sites using Tandem Repeats Finder (version 4.10.0) with default parameters. We chose repeats that meet the following requirements: (a) consensus size between 70 and 250 bp, (b) copy number more than 40, (c) percent matches larger than 98, (d) percent indels less than 2. Seven repeats were generated while two of them were actually the same. Then, template sequences were extracted for each repeat and primers were designed using primer-BLAST. Specificity was checked for all primer pairs to exclude unexpected amplification products. At last, four primers pairs were selected as good candidates for the following experiments.

We also focused on the mitochondria genome for its quantification advantage. We downloaded all FASTA outputs with key words in NCBI, which were *S. japonicum* mitochondrion complete genome, 147 sequences were generated and multi-alignment was done with MAFFT. The result showed that mitochondria genomes have high similarity and we just chose one (JQ781208.1) for primer design. Specificity for all primer pairs were also checked with NCBI primer-BLAST and the candidates were exclusively targeting on *S. japonicum* mitochondrion genome with product size ranging from 60 to 200 base pairs. We finally selected six candidates of the mitochondrion genome. In our study, we initially screened ten pairs of candidate primers, which included four pairs of tandem primers and six pairs of mitochondrial gene primers (Table 1).

**Table 1:** The primers of different target genes

Scafold/genome	Region/genes	Forward primer	Reverse primer	Product size
SKCS01001135.1	*TD-1*	GGGGCACGAGGTGTATGAAA	GGTATAGTGACGCAGC	82 bp
SKCS01000032.1	*TD-2*	GCACGACATTGCCCCCTATTTTT	TTCCCCATTTAAAGATGATGCGAG	90 bp
SKCS01001415.1	*TD-3*	ACTCATCACCGCCAATCAAC	TGCACAACCTTCTTCCCCATA	72 bp
SKCS01000951.1	*TD-4*	AAGGCCGATAGTTTACGCGA	AGGTGACAGGGTTTGCTTCTT	114 bp
JQ781208.1	*COX3*	GCTATGCGTGCTCCTACTCC	TTCAGACCCCAGAAGCAACC	70 bp
	*CYTB*	TGAGCTGCTACTGTATTGACTTCT	ACACGAATAAGCGTATCAACCA	116 bp
	*COX1(1)*	CGTGCGGCTGATCCAATACT	CCCAGTAACACCACCAACTGT	72 bp
	*ATP6*	ATACCAGTGGGTTGTCCGAT	ACGGACGGACCAATAAAACGA	97 bp
	*COX1(2)*	ATGTGCTGTCTTTGGGTTCT	TGGCAACCCATGAACACCTA	176 bp
	*ND5*	AAGCTATGCGTGCTCCTACTC	CAGACCCCAGAAGCAACCAA	70 bp
	*SjR2*	CCATCGATATCTGCTGCATCTC	CAAGGTGAAGTCAAGCGAATGA	
	*ITS2*	TGATGCGCTCGTATATATGAATGC	GGACTAGGCTGTGGTCGGATT	
	*CYTB*	TGAGCTGCTACTGTATTGACTTCT	ACACGAATAAGCGTATCAACCA	

The expression of candidate genes in *S. japonicum*-infected mouse liver

**Fig. 2:**
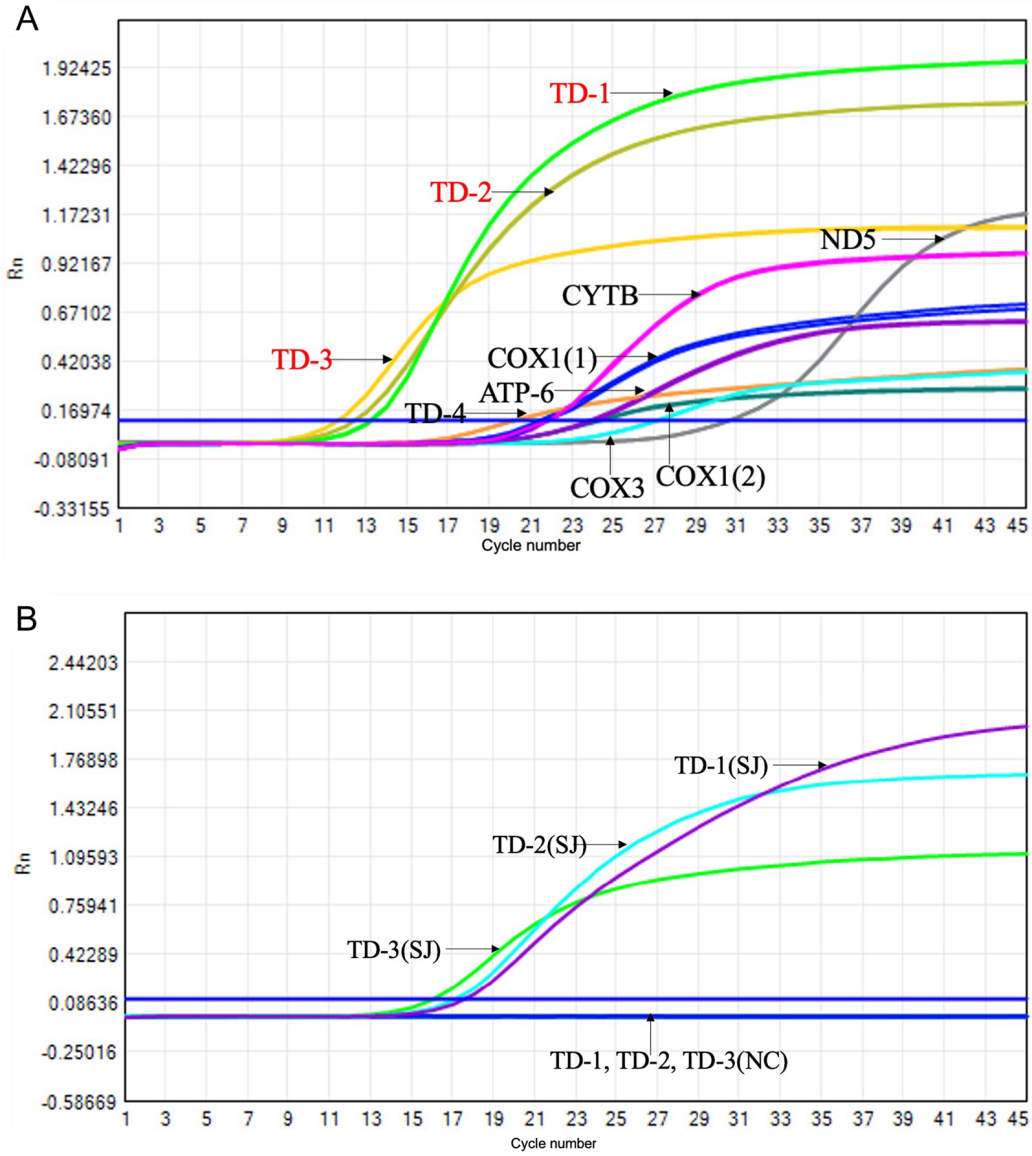
PCR amplification of targets for diagnosis of *Schistosomiasis japonicum*. **A** we examined the expression of four tandem sequences (*TD-1*, *TD-2*, *TD-3*, and *TD-4*) and six mitochondrial genes (*COX1(1)*, *COX1(2)*, *CYTB*,* ATP6*, *COX3*, and *ND5*) in the liver of *S. japonicum*-infected mice at 8 weeks. **B** In both livers of normal mice (NC) and *S. japonicum*-infected mice (SJ) at 8 weeks, we detected the expression of *TD-1*, *TD-2*, and *TD-3*

**Fig. 3:**
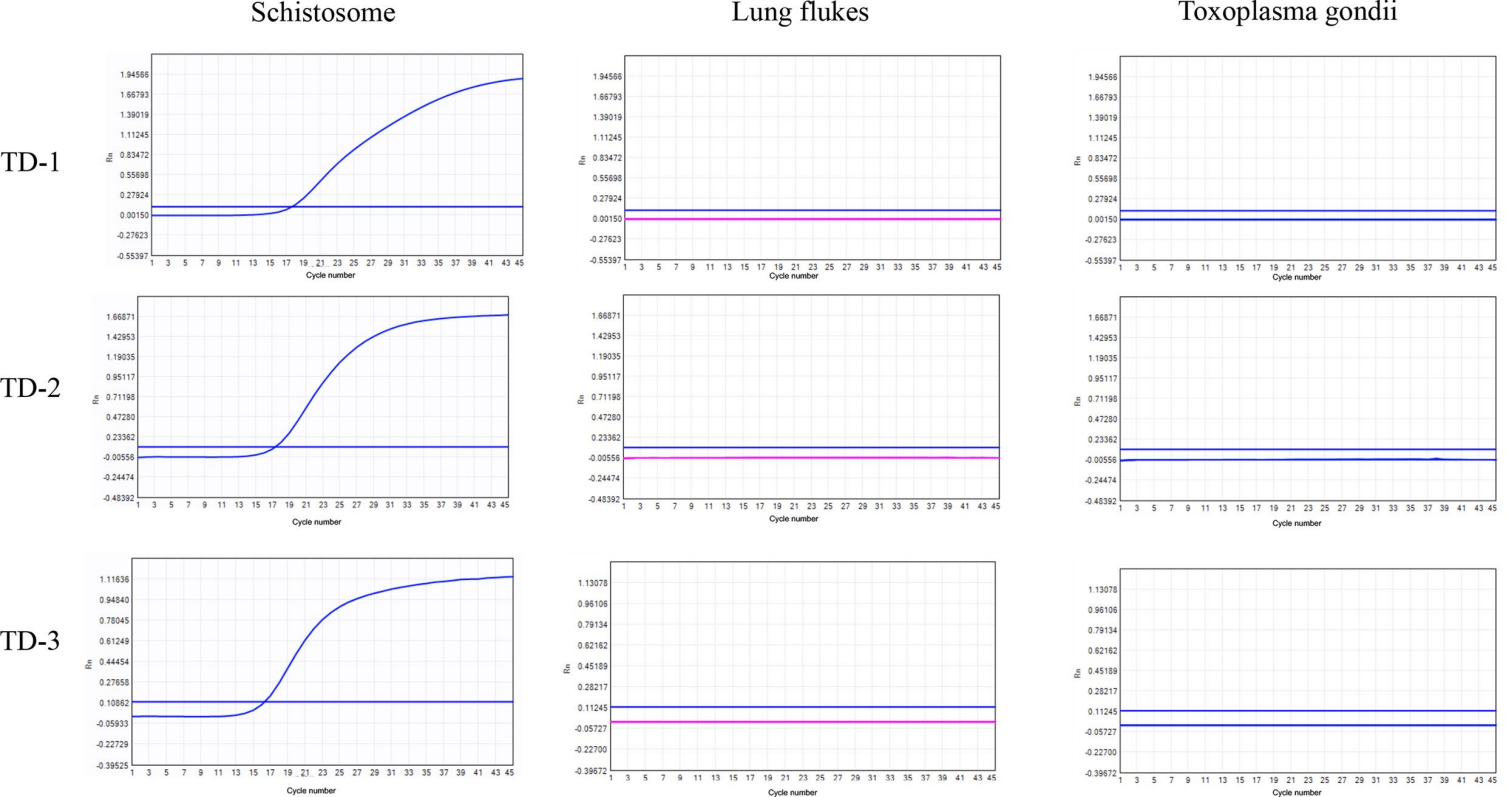
PCR amplification of targets in schistosome, lung flukes, and *Toxoplasma gondii*. To explore the specificity of *TD-1*, *TD-2*, and *TD-3*, we detect our screened targets (*TD-1*, *TD-2*, and *TD-3*) in other parasites, including lung flukes and *Toxoplasma gondii*.

**Fig. 4:**
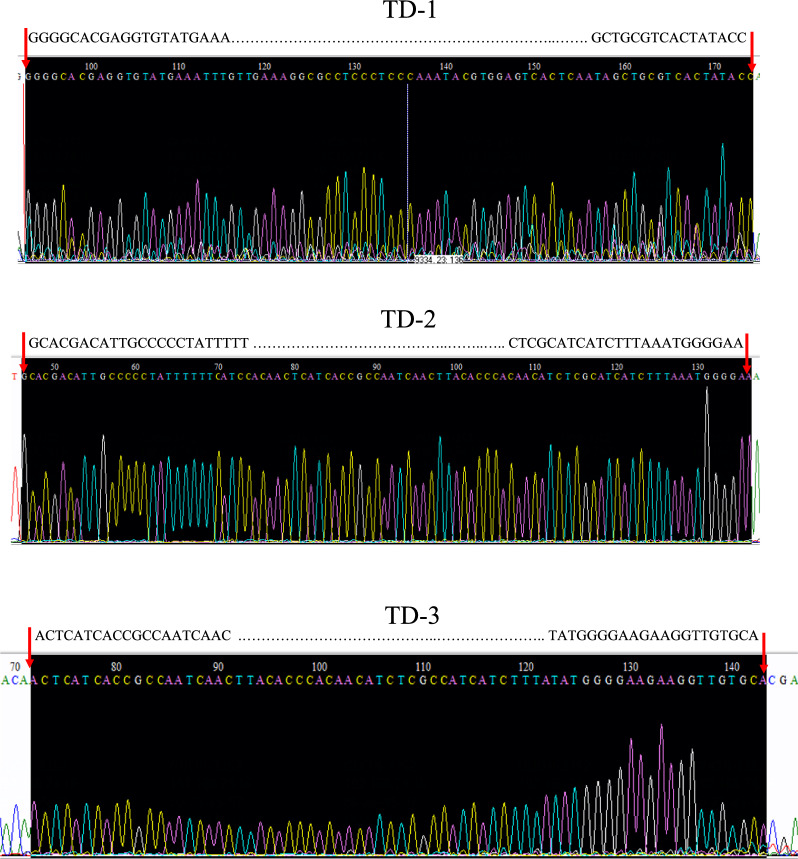
The sequence of Sanger sequencing about *TD-1*, *TD-2*, and *TD-3*. To verify the specificity of the *TD-1*, *TD-2*, and *TD-3* sequences, we sequenced the amplified DNA and verified the *TD-1*, *TD-2*, and *TD-3* sequences by Sanger sequencing

We further investigated the expression of different candidate genes in *S. japonica*. Mice infected with cercariae had accumulated a large amount of eggs in the liver at week 8. We examined the expression of ten candidate genes related to *S. japonicum* in the liver at week 8. As shown in Fig. 2A, the expression of tandem sequences was significantly higher than that of the mitochondrial genes. Meanwhile, the expression of *TD-1*, *TD-2*, and *TD-3* were significantly higher than the expression of *TD-4 *or any other tandem genes, which indicated that *TD-1*, *TD-2*, and *TD-3* might have higher sensitivity for the diagnosis of *S. japonica*. Meanwhile, to explore the specificity of *TD-1*, *TD-2*, and *TD-3*, we tested the expression of the target in the livers of schistosomiasis mice and normal mice. Our results revealed that *TD-1*, *TD-2*, and *TD-3* were detected in the livers of schistosomiasis, whereas they were not detected in the normal mouse livers (Fig. 2B). We further investigated the expression of targets in other parasites, and the results revealed that *TD-1*, *TD-2*, and *TD-3* are not shown in lung flukes and *Toxoplasma gondii* (Fig. 3). To verify the sequences of *TD-1*, *TD-2*, and *TD-3*, we sequenced the amplified DNA and verified the sequences of *TD-1*, *TD-2*, and *TD-3* by Sanger sequencing (Fig. 4).

### Detection of *S. japonicum* cfDNA in infected mice at different weeks

**Fig. 5:**
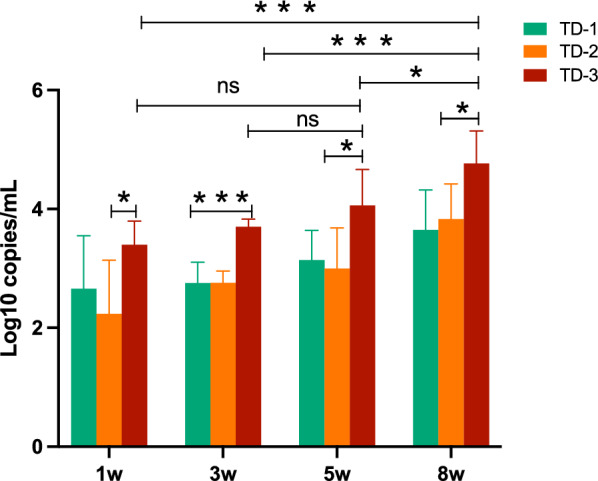
Detection of cfDNA (*TD-1*, *TD-2*, and *TD-3*) in infected mice at different weeks. After extracting cfDNA from plasma of *S. japonicum*-infected mice at 1 week, 3 week, 5 week, and 8 week (*n* = 5), we detected the expression of *TD-1*, *TD-2*, and *TD-3* (*n* = 5). The significance level was tested by unpaired *t*-test and the data are shown as the mean ± s.d. values (*p < 0.05, **p < 0.01, ***p < 0.001, ****p < 0.0001)

**Fig. 6:**
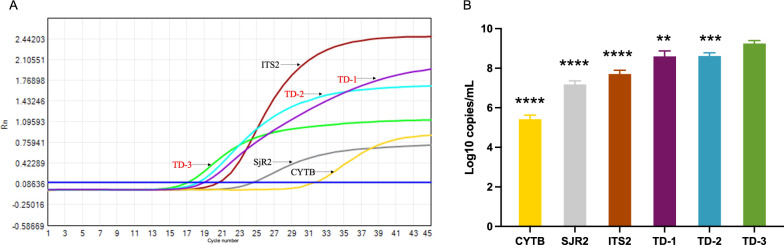
Detection of our targets and other reported targets. **A** The plot of PCR amplification of *TD-1*, *TD-2*, *TD-3*,* SjR2*, *ITS2*, and *CYTB* in the livers of *S. japonicum*-infected mice at 8 weeks. **B** The expression level comparison between *TD-3* and other targets in the livers of *S. japonicum*-infected mice at 8 weeks (*n* = 5). The significance level was tested by unpaired *t*-test and the data are shown as the mean ± s.d. values (*p < 0.05, **p < 0.01, ***p < 0.001, ****p < 0.0001)

The cercariae can develop into adult worms after infecting the host, which could produce a large number of eggs deposited in liver. Some studies have suggested that adult schistosomes could survive in the host for decades without treatment. Adult worms can be treated with drugs, but there is currently no effective method to eliminate the deposited eggs, which development into chronic schistosomiasis. Around the fifth week after infection of cercariae, eggs start to be produced, and a large number of eggs are present in the liver by the eighth week. We established a mouse model of *S. japonica* and collected mouse plasma at 1 week, 3 week, 5 week, and 8 week after infection. After extracting cfDNA from plasma, we detected the expression of *TD-1*, *TD-2*, and *TD-3* in peripheral blood. As shown in Fig. 5, all of the three targets could be detected at different time points. There was no significant difference in expression levels of *TD-1* and *TD-2*, but the expression level of *TD-3* was significantly higher than that of *TD-1* and* TD-2* at different time points. Meanwhile, there was no significant difference in the expression of TD-3 at week 1, week 3, and week 5, but the level of *TD-3* at week 8 was significantly higher than that at other time points. Our results indicated that *TD-3* might be a sensitive diagnostic target for *S. japonica*. Previous research has also reported other targets in *S. japonica* such as *SjR2*, *ITS2* and *CYTB*. Therefore, we extracted DNA from the livers of schistosome-infected mice and tested for *TD2*, *TD3*, *SjR2*,* ITS2*, and* CYTB*. The results showed that *TD3* remains the most highly expressed target among them (Fig. 6A, B).

### Detection of *S. japonicum* cfDNA in different number of cercariae infection

**Fig. 7:**
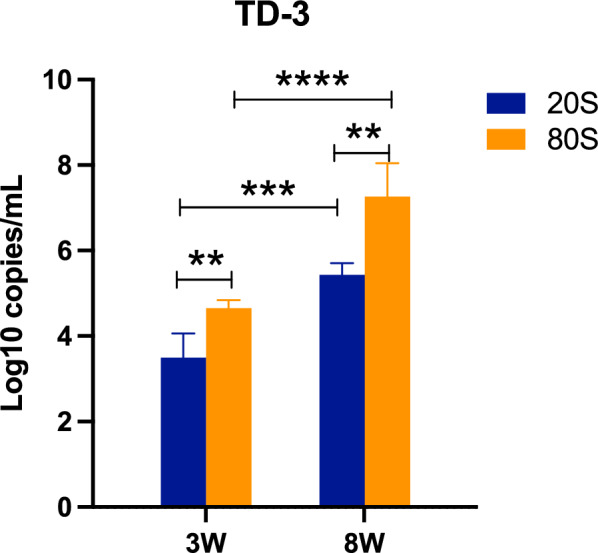
Detection of cfDNA (*TD-3*) in different number of cercariae infection. We infected mice with different quantities of cercariae (20 s and 80 s) and detected the levels of cfDNA (*TD-3*) at three weeks and eight weeks after cercariae infection(*n* = 5). The significance level was tested by unpaired *t*-test and the data are shown as the mean ± s.d. values (*p < 0.05, **p < 0.01, ***p < 0.001, ****p < 0.0001)

To determine whether the level of cfDNA is related to the severity of cercariae infection and disease progression, we infected mice with different quantities of cercariae (20 s and 80 s). According to the schistosome life cycle, the first 3 weeks postinfection primarily involve the development of cercariae into adult worms. Notably, the detection levels of *TD-3 *were significantly higher in the 80 s infection group compared with the 20 s infection group (Fig. 7). By the eighth week postinfection, schistosomes had produced a substantial number of eggs in liver, and *TD-3* levels were also higher in the 80 s infection group than in the 20 s infection group. Furthermore, regardless of the initial infection intensity (20 s or 80 s), *TD-3* levels at the eighth week were higher than those at the third week (Fig. 7). These results suggest that the more cercariae the host is infected with, the higher the peripheral *TD-3* levels detected. Additionally, as egg deposition increases, peripheral *TD-3* levels also rise.

### Expression of *TD-3* in patients with *Schistosomiasis japonica*

We further investigated the diagnostic value of *TD-3* in patients with *S. japonica*. We enrolled patients with chronic *S. japonica* (*n* = 42) and healthy volunteers (*n* = 8). The patients we included were those who were previously diagnosed with *S. japonica*, admitted to the hospital and treated with praziquantel. We extracted circulating cfDNA from all individuals. Our results showed that *TD-3* was not detected in the 8 healthy volunteers but was detected in 9 of 42 patients (21.43%) in peripheral blood. There were no significant differences in age, gender, white blood cell (WBC), hemoglobin (HB), platelet (PLT), total bilirubin, alanine aminotransferase (ALT), aspartate aminotransferase (AST), albumin, gamma-glutamyl transferase (GGT), and IgG between positive patients and negative patients for *TD-3* (Table 2). Although there was no statistical significance, it was observed that 44.44% of positive patients (*TD-3*) displayed reticular changes observed by liver ultrasounds, whereas only 21.21% of negative patients (*TD-3*) showed such changes. Additionally, 33.33% of positive patients (*TD-3*) presented with ascites, while only 15.15% of negative patients (*TD-3*) exhibited the same symptom.

**Table 2:** The characteristics of enrolled patients

Characteristics	*TD-3* negative patients (*n* = 33)	*TD-3* positive patients (*n* = 9)	*p*-Value
Age (in years)	63.64 (9.89)	63.67 (9.80)	0.9699
Male	25 (75.76%)	7 (77.78%)	0.7525
WBC (×10^9^/L)	6.31 (2.28)	4.90 (1.00)	0.0554
HB (g/L)	142.76 (18.13)	140.78 (33.30)	0.8386
PLT (×10^9^/L)	205.61 (71.97)	165.56 (71.01)	0.1174
Total bilirubin (µmol/L)	15.39 (10.67)	15.21 (7.45)	0.8985
Albumin(g/L)	43.54 (3.32)	41.59 (7.52)	0.7804
ALT (U/L)	30.00 (27.92)	22.80 (12.73)	0.5302
AST (U/L)	31.73 (44.72)	24.83 (10.43)	0.7799
GGT (U/L)	36.36 (25.51)	69.22 (82.06)	0.2270
IgG positive^#1^	7 (21.21%)	1 (11.11%)	0.8374
Reticular changes^#2^	7 (21.21%)	4 (44.44%)	0.3283
Ascites	5 (15.15%)	3 (33.33%)	0.4518

WBC, white blood cell; HB, hemoglobin; PLT, platelet; ALT, alanine aminotransferase; AST, aspartate aminotransferase, GGT, gamma-glutamyl transferase

^#^1 Anti-schistosoma IgG was detected in serum of patients with schistosomiasis

^#^2 Reticular changes is an unique fibrotic pattern of liver in ultrasonography about schistosomiasis

## Discussion

Schistosomiasis remains an important public health problem that threatens human life and health [6]. Early diagnosis and treatment of schistosomiasis can greatly improve survival and prognosis of patients. Although there are various methods for diagnosing of schistosomiasis, many limitations are still exist [19]. Liquid biopsy is an novel diagnostic technology that can diagnose diseases through bodily fluids such as blood, urine, and saliva [20, 21]. Currently, liquid biopsy has been applied to various disease such as tumors, transplantation, and pregnancy, achieving obvious breakthroughs and significance [22–25]. Meanwhile, the techniques for extracting and enriching cfDNA from peripheral blood are also constantly improving. In this study, we evaluated the diagnostic value of circulating cfDNA for *Schistosomiasis japonica*.

Screening appropriate targets is beneficial to improve the sensitivity and specificity of diagnosis. We are interested in tandem sequences and mitochondrial genes of *S. japonicum*. Tandem sequences are highly repetitive sequences that can improve the sensitivity of diagnosis [26, 27]. Mitochondrial genes are highly conserved and widely used in the diagnosis of diseases [28–30]. Through data screening and analysis, we ultimately obtained four pairs of tandem primers and six pairs of mitochondrial gene primers. We detected the expression of candidate genes in the mouse liver with schistosome egg deposition. The expression of the tandem candidates was significantly higher than that of the mitochondrial genes, which indicate that the tandem candidates might have better sensitivity for the diagnosis of *S. japonica*. We further validated the specificity of tandem candidates by testing for nonexpression on normal liver and other parasite samples such as lung flukes and *Toxoplasma gondii*. Meanwhile, we have selected top three highly expressed tandem candidates (*TD-1*, *TD-2*, and *TD-3*) and sequenced the amplified cDNA for validation.

Currently, there are still some problems in the diagnosis of acute schistosomiasis patients. The detection of schistosome eggs or schistosome-specific antibodies using microscopy or immunological methods accounts for the majority of current diagnostic approaches [31–33]; however, it takes about 4–6 weeks to produce eggs after infection [19]. Meanwhile, schistosome antibodies might be preexisting in the body of patients who have been previously infected, and the detection of antibodies might not be an effective and ideal indicator for diagnosis of reinfection [34]. Failure to diagnose early would delay treatment for patients, especially for those with less symptoms. The adult schistosomes could be effectively treated, but the deposition of their eggs in tissues would cause granuloma formation and fibrosis, resulting in irreversible damage [1, 35]. After infecting mice with cercariae, we extracted cfDNA from the peripheral blood of mice and detected the expression of *TD-1*, *TD-2*, and *TD-3* of *S. japonicum*. Our results showed that we could diagnose infection of *S. japonicum* through circulating cfDNA as early as the first week after infection. We measured the expression levels of circulating cfDNA (*TD-1*, *TD-2*, and *TD-3*) at different time points and found that *TD-3* expression was significantly higher than that of *TD-1* and *TD-2*. Additionally, the expression levels of *TD-1*, *TD-2*, and *TD-3* have increased significantly with the production of schistosome eggs. Our results show that early diagnosis of schistosome infection could be achieved by detecting circulating cfDNA. The homogeneous distribution of cfDNA in peripheral blood could promote the stability of detection. Meanwhile, to compare our targets with those reported targets such as *SjR2*, *ITS2*, and *CYTB*, we further tested their expression in the livers of schistosome-infected mice. Our results indicate that the expression level of *TD-3* is significantly higher than that of other targets, suggesting that *TD-3* might have higher sensitivity in diagnosing *S. japonica*.

Schistosomiasis primarily consists of two stages. One is acute schistosomiasis caused by cercariae infecting the host, and the severity of the disease often correlates with the number of cercariae infections. The other involves the formation of granulomas and liver fibrosis due to egg deposition by adult worms. To explore whether cfDNA is associated with cercariae infection differences and egg deposition, we infected mice with different quantities of cercariae. The results showed that the level of cfDNA (*TD-3*) in the 80 s infection group was significantly higher than in the 20 s infection group. Additionally, cfDNA (*TD-3*) levels increased after egg deposition. Our results indicated that early diagnosis of *S. japonica* could be achieved through the detection of cfDNA (*TD-3*), and there was a certain correlation between the levels of cfDNA (*TD-3*) and cercariae infection severity. Early detection and diagnosis of schistosomiasis are crucial for patient treatment and improving prognosis.

Chronic schistosomiasis is mainly caused by the deposition of schistosome eggs in tissues, and the severity of schistosomiasis is often closely related to the accumulation of schistosome eggs [36]. However, there is a lack of effective approaches and indicators to assess the severity of schistosomiasis. We tested 42 patients with chronic *S. japonica* and circulating cfDNA (*TD-3*) was detected in nine patients. There is an increasing trend in reticular changes of liver ultrasounds and ascites in *TD-3* positive patients compared with *TD-3* negative patients, which need to more data for validation. For patients with chronic schistosomiasis, eggs might be destroyed and even disappear after praziquantel treatment and a long course of the disease, and the granulomas is then replaced by fibrous tissue. For those patients, it is difficult to detect cfDNA. However, for schistosomiasis patients who could still be detected with cfDNA, we should be vigilant about whether they have reinfection with cercariae or whether there are still adult worms and egg production in their bodies. For patients who test positive for cfDNA, we should pay more attention to changes in their condition and provide effective treatment promptly.

## Conclusions

In our ﻿study, we have screened highly sensitive targets for the diagnosis of *S. japonica*, and the detection of circulating cfDNA is a rapid and effective method for the diagnosis of *S. japonica*. However, there are still some limitations in our study. We have not validated whether this target is suitable for diagnosing other species of schistosomiasis. Our targets need further clinical data validation. In summary, detecting cfDNA might be an important method for monitoring schistosomiasis.

### Supplementary Information

**Additional file 1: Figure S1.** The standard curves and the lowest limit of detection for *TD-1*, *TD-2*, and *TD-3*.

## Data Availability

All of data and materials are available.
