# Supplementary material for: Circulating cell-free DNA as a biomarker for diagnosis of Schistosomiasis japonica
Source: Parasit Vectors. 2024 Mar 6;17:114. doi: 10.1186/s13071-024-06203-x (PMC10918879; doi:10.1186/s13071-024-06203-x)
Supplement: Supplementary file 1 — Additional file 1: Figure S1. The standard curves and the lowest limit of detection for TD-1, TD-2, and TD-3. [file 13071_2024_6203_MOESM1_ESM.docx]

Additional file 1: Figure S1. The standard curves and the lowest limit of detection for TD-1, TD-2, and TD-3.

| TD-1 | | TD-2 | | TD-3 | |
| --- | --- | --- | --- | --- | --- |
| Concentration (copies/mL) | Positive rate(%) | Concentration (copies/mL) | Positive rate(%) | Concentration (copies/mL) | Positive rate(%) |
| 200 | 70 | 200 | 90 | 200 | 75 |
| 300 | 95 | 300 | 90 | 300 | 100 |
| 400 | 95 | 400 | 100 | 400 | 100 |
| 500 | 100 | 500 | 100 | 500 | 100 |

We diluted the predetermined plasmids of TD-1, TD-2, and TD-3 with negative serum samples to concentrations of 500 copies/mL, 400 copies/mL, 300 copies/mL, and 200 copies/mL. We then extracted DNA from various quality control samples and performed 20 repeated detections for each concentration. Using SPSS software, we conducted probit analysis to calculate the 95% confidence intervals. The lowest limit of detection were as follows: TD-1(351 copies/mL), TD-2(300 copies/mL) and TD-3(261 copies/mL).
